# Aggressive Management of Tibial Osteomyelitis Shows Good Functional Outcomes

**Published:** 2011-01-25

**Authors:** Raewyn Campbell, M. G. Berry, Anand Deva, Ian A. Harris

**Affiliations:** Departments of Plastic and Reconstructive Surgery and Orthopaedic Surgery, Liverpool Hospital and the South Western Clinical School, University of New South Wales, Sydney Australia

## Abstract

**Background:** Severe open tibial fractures can be successfully treated acutely with a combined orthopedic and plastic surgery approach, but a proportion will go on to develop chronic osteomyelitis. For the past 6 years, an aggressive approach of bone and soft tissue debridement followed by skeletal reconstruction and vascularized tissue transfer has been pursued by the orthopedic and plastic surgery teams at Liverpool Hospital. We present the results of our patient series. **Methods:** All patients treated for chronic osteomyelitis by combined skeletal stabilization, debridement, and flap coverage between January 2000 and July 2006 were included. Clinical record review was combined with patient interviews and questionnaires. Outcome measures included fracture union, stable soft tissue coverage, freedom from infection, mobility, return to work/sport, and pain. **Results:** Twelve patients were followed up after a mean of 4.2 years. Patients had undergone a mean of 8.4 procedures prior to treatment, and a mean of 2.5 procedures as part of their treatment. We achieved fracture union, stable soft tissue coverage, and eradicated infection in all patients. All patients were walking, 10 unaided, and 80% had returned to work. All but one patient involved in sport at the time of injury had returned to sport. Two patients had mild pain when walking long distances only. **Conclusion:** Skeletal stabilization, debridement, and flap coverage is costly and complex surgery. However, in our series, these interventions resulted in eradication of infection and good clinical outcomes in most cases, providing an alternative to both amputation and long-term antibiotic therapy.

Open tibial fractures carry a 4.5% to 20% reported incidence of osteomyelitis (OM)^1^^-^7 which correlates directly with injury severity.^1^,^3^,^8^ The most common causes of posttraumatic OM are “… retained necrotic and infected bone, avascular or infected scar, dead space and inadequate skin cover”^9^ and chronic granulation tissue in the medullary canal.^10^ Once established, posttraumatic tibial OM is difficult to treat with reported failure rates of up to 30%.^11^^-^13 Treatment options at this stage include amputation or limb salvage. The principles of salvage involve aggressive resection of infected skeletal and soft tissues thereby necessitating a multidisciplinary approach to reconstruct potentially large defects.^13^

To date, there have been few reports of the long-term functional and quality-of-life outcomes following an aggressive limb salvage approach.^14^,^15^ In addition, there is little standardization of patient populations and indications for the differing treatment options, which vary with the experience and expertise of the treating teams.^15^

Our study aimed to analyze the acute and intermediate-term functional outcomes following an aggressive, multidisciplinary treatment program at a tertiary referral center and major trauma unit and to provide data on its therapeutic efficacy for chronic tibial OM.

## MATERIALS AND METHODS

A review of the surgical database and clinical charts from January 2000 to July 2006 was performed. Inclusion criteria included all patients 18 years and older with radiologically (lytic lesions, bone resorption, bone sequesters, sclerosis, and/or soft tissue swelling) and microbiologically proven OM of greater than 6 weeks' duration in a posttraumatic tibia fracture presenting to our center for treatment. Patients were contacted for follow-up interviews, clinical assessment, and questionnaires at a minimum of 2 years posttreatment. Patients who had not completed treatment at follow-up (eg, Ilizarov frame still in situ) were excluded.

Outcome measures included operative success (radiologically confirmed bony union and stable soft tissue cover), freedom from infection (based on hematological, microbiological, clinical, and, where performed, nuclear medicine investigations), mobility, return to work, return to sport, and residual pain.

The Lower Extremity Functional Scale (LEFS)^16^ was used to assess the activities of daily living (ADLs). The LEFS is a self-reported questionnaire comprising 20 functional leg activities each scored on a 5-point ordinal scale, from 0 (extremely difficult/impossible to perform) to 4 (no difficulty). It is scored out of a maximum of 80 to allow functional ability to be expressed nominally. The reliability, construct validity, and sensitivity to clinical change of the LEFS has been shown to be superior to other functional outcome scales in prospective, multicenter trials.^16^,^17^

Ethics approval was obtained from the local area health service human research ethics committee.

## RESULTS

Of 16 106 trauma admissions to our center over this period, 3598 had lower limb trauma. Of these, 12 patients matched inclusion criteria for the study and all consented to inclusion (Tables 1 and 2). The mean follow-up was 4.2 years (range, 2.3–6.0 years), and all subjects were successfully contacted for contemporaneous review.

### Pretreatment parameters

Tables 1 and 2 summarize the etiology and patient demographics for our subjects. The mean age at injury was 39 years (range, 19-63 years). Injuries were from motor vehicle/motorbike accidents in 33.3%, industrial injuries in 33.3%, and falls in 16.7%. One patient's injury was sustained during an assault and another from a fall between a train and the station platform. This patient underwent a below knee amputation (BKA) on the contralateral leg on the day of injury. Six patients sustained Gustilo^6^,^18^ class IIIB injuries, 2 sustained Gustilo II, and 4 patients had closed fractures. Seven patients sustained additional injuries at the time of initial injury and 9 patients had comorbidities (including smoking, diabetes, hepatic cirrhosis, multiple antibiotic allergies, hypothyroidism, and deep vein thrombosis) some of which classified them as physiologic class B-hosts using the Cierny Mader classification system.^19^ Distal fractures were the most common (*n* = 6), followed by proximal (*n* = 4) and 2 mid-shaft fractures. Mean duration of diagnosed OM was 32 months (range, 2–216).

### Operative details

**Figure 1:**
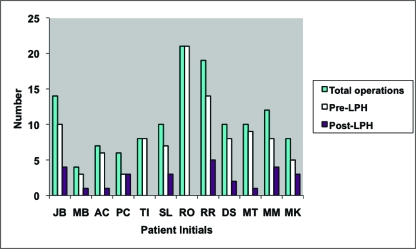
Number of surgical procedures prior to treatment at LPH and number of procedures at LPH.

**Figure 2:**
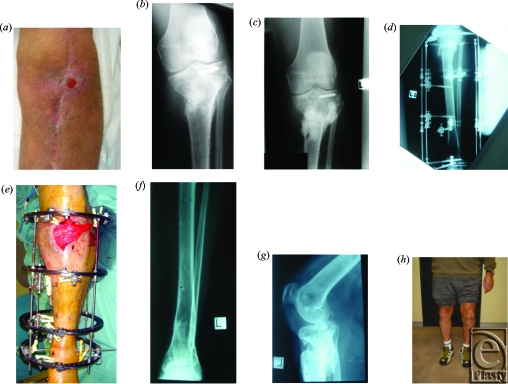
(*a*) Patient with chronic tibial OM and discharging sinus. (*b*) x-ray after removal of initial internal fixation and prior to debridement, demonstrating proximal tibial OM. (*c*) After debridement and insertion of antibiotic beads. (*d*) Osteotomy and application of Ilizarov frame. (*e*) Ilizarov frame and gastrocnemius flap. (*f*) Final result of distal tibial osteotomy site 10 months post-removal of Ilizarov frame. (*g*) Final result of proximal tibial site after bone grafting and removal of Ilizarov frame. (*h*) The final clinical result.

Patients underwent a mean of 11.4 (range, 4–21) procedures over 6.3 (range, 3–11) hospital admissions to achieve both bony union and stable wound closure. Patients underwent a mean of 8.4 procedures prior to presentation to our unit, and a mean of 2.5 procedures were performed at our institution (Fig 1). Debridement and soft tissue coverage was performed in a staged manner in 10 patients and during a single session in 2. These single sessions were, however, followed by at least one further operation, either to remove the Ilizarov frame and/or to debulk a free muscle flap. Procedures comprised debridement, external/internal fixation, bone grafting, and soft tissue flaps (Fig 2).

The majority of patients underwent Ilizarov frame application or external fixation via other means (75%). Four patients were treated with 5 free flaps (2 latissimus dorsi, 1 rectus abdominis, 1 anterolateral thigh, and 1 gracilis-free flap). One patient underwent 2 successive free flap reconstructions following failure of the first flap on day 4 due to deep venous thrombosis. The remaining 8 patients had soft tissue reconstruction using either pedicled muscle (*n* = 4, gastrocnemius) or fasciocutaneous flaps (*n* = 4). All patients received thromboprophylaxis in the form of subcutaneous low molecular weight heparin and graduated compression stockings during inpatient stays and as outpatients where appropriate. Intermittent pneumatic compression devices were used intraoperatively and throughout the periods of immobility.

### Microbiological investigations

The mean total duration of antibiotics was 12.8 months (range, 1.8-33). The predominant organisms cultured were Staphylococcus species (*n* = 9, S. Aureus, including methicillin-resistant strains) and Pseudomonas species (*n* = 3). Five patients had polymicrobial infections (Table 3).

### Long-term outcomes

All patients had radiologically confirmed successful bony union and stable soft tissue cover and were free of infection at follow-up (based on hematological, microbiological, and/or nuclear medicine investigations).

All patients are currently walking. Two patients require some simple form of ambulatory aid (one of whom underwent an immediate/acute BKA on the contralateral leg and now mobilizes independently using a walking stick for long distances only). Two of our patients reported pain, which was mild (score 1/10) and occurred after walking long distances only. Seven patients had associated injuries, and in 6 patients, these injuries affected their rehabilitation. These injuries included fractured scaphoid bone, fractured ribs, dislocated glenohumeral and acromioclavicular joint, vertebral fractures, fractured metatarsal bone, and a fractured radius. One patient underwent a delayed BKA of the affected limb for *distal* nonunion of a fracture sustained in a separate incident 1 year after the original injury (a *proximal* fracture from a high-speed motor vehicle accident) and 6 months after the removal of the Ilizarov frame. This distal fracture was not in the location of a pin site and was not osteomyelitic, unlike that of the proximal tibia, which was successfully treated, avoiding an above knee amputation. Furthermore, amputation was discussed with this patient at the time of the original, proximal tibial injury due to its severity (Gustilo IIIB with gross contamination) but was declined in favor of attempted salvage. All surgery subsequent to the removal of the Ilizarov frame involved the BKA and the treatment of complications caused by a poorly fitting prosthesis. This patient achieved the lowest LEFS (17.5). He now mobilizes independently with a prosthesis and no other mobility aids.

Eight of the 10 patients working at the time of injury have returned to work, all to their preinjury occupations, although 3 of these patients now work in more sedentary roles. The remaining 2 previously working patients have retired and receive a disability pension as a result of their injury. Two patients were already retired at the time of injury. All 4 patients involved in sport prior to their injury have returned to sport (1 patient at the preinjury level, the remainder at a modified level). The average LEFS score was 51 (14–80) representing an average of 64% (range, 18-100) of maximal function.

During an average follow-up of 4.2 years, there has been a 100% operative success rate with all patients having radiological and clinical confirmation of bony union and stable soft tissue cover (including the site of OM in the patient who underwent a delayed BKA). All patients were walking and were free from infection at the time of study and had minimal to no pain during ADLs. No patients have expressed either regret at pursuing a reconstructive path, or a desire for amputation.

## DISCUSSION

This study aimed to assess the technical and functional outcomes of an aggressive, multidisciplinary approach to the treatment of tibial OM. Our findings demonstrate that this approach, based at a tertiary referral center and utilizing the expertise of experienced surgical, medical, and allied health departments, can successfully achieve functional limb salvage and freedom from infection. During a mean follow-up of 4.2 years and after a mean of 2.5 surgical procedures at our institution, 100% of our patients were free from infection with stable bony union and soft tissue cover. These results compare favorably with those in the recent literature.^11^,^20^,^21^ Furthermore, 100% of our patients were mobilizing, 83% independently. These results also compare similarly to those of Siegel et al^20^ who reported an 85% independent mobility outcome in 46 patients who underwent limb salvage surgery for chronic tibial OM over an 18-month period. All of our patients playing sport prior to injury have returned to sport, compared to 63% rate of return to sport in the studies of Siegel et al.^20^ Interestingly, 17% of our patients reported pain. This compares with 89% of the patients of Siegel et al. These differences may be explained by the slightly different patient population, as all of the patients of Siegel et al had soft tissue coverage with free or rotation flaps, whereas only two thirds of our patients had either free or rotation flaps, the remainder had fasciocutaneous flaps.

Overall, our patients reported a return to a mean of 64% of lower extremity function, with 67% performing ADLs with minimal to no difficulty, 25% reporting moderate difficulty, and 1 patient reporting severe difficulty. This patient was the single amputation in our series as discussed earlier.

Fortunately, chronic OM is relatively uncommon; hence the low patient number in this study (12 of 3598 patients with lower limb trauma). Furthermore, despite an empirical understanding and the existence of many objective criteria (eg, evidence of infection for >6 weeks), chronic OM is difficult to define as no widely accepted definition exists.^14^

Chronic tibial OM is particularly difficult to manage and treatment fails in 10% to 30% of cases.^5^,^11^,^14^,^22^,^23^ Furthermore, it has the worst prognosis of any bone,^15^ the potential for malignant transformation,^24^ and can recur at any time (although most commonly within 2 years).^11^,^23^ The tibia is particularly prone to OM due to its large subcutaneous anterior surface and scant muscle coverage providing minimal protection and blood supply.^25^ The most common pathogens involved are coagulase-positive staphylococci^1^ and gram-negative bacilli,^14^ as was the case in this study (Table 3). Furthermore, chronic OM is frequently polymicrobial,^14^ a result confirmed in 42% of the patients in our study.

Treatment of tibial OM is intensive and demanding on patients, staff, and resources, as is evidenced by the large number of previously unsuccessful interventions and the duration of OM in our patient population. The importance of a successful outcome in the treatment of chronic OM of the lower limb is further highlighted by Lerner et al,^26^ who found that patients with chronic refractory OM scored lowest in quality of life parameters compared with patients with long bone fracture nonunion and those who had undergone posttraumatic amputation. Future developments, such as the use of bone morphogenic protein in lieu of bone grafts, may further improve the treatment of this condition.^27^

Limb salvage surgery has traditionally been found to require more operative procedures, a longer hospital stay, and rehabilitation process^28^^-^31 and to be more expensive when compared with amputation.^29^,^31^^-^33 It has been calculated that walking with crutches on a functionless salvaged leg requires 15% more energy than walking with a below-knee prosthesis.^34^ On the contrary, most studies comparing the costs of salvage versus amputation do not account for the recurring long-term costs of lower limb prostheses, focusing only on the acute costs of hospitalization.^29^,^31^^-^33 Despite potentially greater *initial* hospital costs and longer rehabilitation, patients with reconstructed limbs have been shown to ultimately represent a lower global cost to the community (including nonhospital costs and pensions).^28^,^34^ They have also been found to have fewer interventions,^28^ similar or better functional outcomes,^28^,^35^,^36^ higher rates of returning to employment,^1^,^35^ similar or improved quality of life ratings,^28^,^37^ and better physical outcome scores^2^,^28^,^36^,^37^ than amputees. Initial costs of newer model prostheses range from AUD2500.00 for a below knee prosthesis^38^ to AUD51,000.00 for an above knee prosthesis with a microprocessor.^39^ Prostheses require regular alterations and replacement of parts and can have a lifespan of only 6 months initially until stump maturity at around 3 years following amputation. Thereafter, prostheses have an average lifespan of 2.25 years. This may be shorter for younger patients who place a greater physical demand on the prosthesis. In a study comparing the cost of amputation with limb salvage using ilizarov reconstruction, Williams^34^ noted that the projected lifetime costs for amputees with prostheses were 7 times that of reconstructed limbs. Amputees are also more likely to abandon sporting activities.^28^ In addition, Francel et al^29^ noted that reconstructees preferred their own limb, recording a 96% satisfaction rate. In a study comparing amputated versus reconstructed patients, Hertel et al^28^ reported both higher body integrity scores in reconstructed patients and significantly less impairment in their nonprofessional lives. They also experienced less social stigmatization, were more likely to return to their preinjury profession, and had a lower incidence of reliance on disability allowances.^28^

It must be acknowledged that our results may be subject to influence by the small cohort and intermediate duration of follow-up. However, OM most commonly recurs within the first 2 years,^11^ and, despite the small cohort, this paper provides one of few functional assessments of patients with chronic OM who have undergone such aggressive treatment. Furthermore, comparing results of the treatment of chronic tibial OM in the literature is difficult due to differing follow-up periods, fracture, and OM classification systems (and the interobserver bias and low predictive value of fracture classification systems^11^ and functional assessment scales, if the latter are used at all.

Our results confirm that amputation is rarely indicated for the treatment of chronic tibial OM, where there is an option of aggressive limb salvage surgery performed by a specialized multidisciplinary team with experience in aggressive debridement, skeletal reconstruction, and vascularized tissue transfer.

## CONCLUSION

Our initial experience with this aggressive approach has been positive and supports the aspirations that limb salvage can ultimately provide a limb that is superior to a prosthesis. Such interventions to allow freedom from infection are costly in the short term to both patients and healthcare providers, but are worthwhile given the alternatives of long-term antibiotic usage and major limb amputation. An aggressive approach returns the majority to work (80%) and sport (100%) and can be associated with 100% operative success as seen in this study.

## Figures and Tables

**Table 1:** Patient demographics*

Patient number, n Male:Female Mean age (range), y	12 9:3 39(19-63)	
Mechanism (no. of patients)	MVA	2
	MBA	2
	Industrial	4
	Fall	2
	Other	2
Class (no. of patients)	Gustillo II	2
	Gustillo IIIb	6
	Closed	4
Additional injuries (no. of patients)	7	

*MBA indicates motorbike accident; MVA, motor vehicle accident.

**Table 2:** Results*

Patient Number	Age at Injury, y/Sex	Admissions	Fracture Location	Total Operations	Antibiotics (months)	Follow-up, y	LEFS (% of Max Function)	Outcome
1†	56/M	11	Proximal	14	13	3.5	26.25	MWA, FFI
2†	55/F	5	Distal	4	18	4.4	100	MI, RTW, FFI
3†‡	21/M	3	Distal	7	7.75	2.75	93.75	MI, RTW, RTS, FFI
4†‡	19/M	6	Distal	6	1.75	5.25	100	MI, RTW, RTS, FFI
5	63/F	3	Midshaft	8	26.25	5	67.5	MWA, FFI
6†‡	26/M	6	Distal	10	12.5	5.5	83.75	MI, RTW, RTS, FFI
7†	23/M	9	Proximal	21	6.63	6	48.75	MI, FFI, RTW
8†	48/M	4	Proximal	19	33	4.9	32.5	MI, FFI
9†	24/F	9	Distal	10	16.75	5.1	76.25	MI, RTW, FFI
10†	38/M	6	Proximal	10	9	3.1	17.5	BKA, MI, RTW, FFI
11†‡	31/M	8	Midshaft	12	2	2.5	62.5	MI, RTW, RTS, FFI
12	61/M	5	Distal	8	4.14	2.3	61.25	MI, FFI
Mean	38.75	6.3		11.4	12.84	4.36	64.17	

*BKA indicates below knee amputation; FFI, free from infection; LEFS, Lower Extremity Functional Scale; MI, mobilising independently; MWA, mobilising with walking aids; RTS, returned to sport; RTW, returned to work.

†Working prior to injury.

‡Playing sport prior to injury.

**Table 3:** Pathogens cultured intraoperatively from patients*

Cultured Pathogen	No. of Patients	Patient No.
Staphylococcus aureus	5	3,4,7,10,11
MRSA*	4	5,6,8,9
Pseudomonas auruginosa	3	8,9,10
Escherichia coli	2	2,12
Staphylococcus epidermis	1	1
Enterococcus faecalis	1	8
Serratia marcescens	1	1
Streptococcus milleri	1	12
Acinetobacter	1	12
Proteus vulgaris	1	12
Proteus mirabilis	1	8
Polymicrobial	5	1,8,9,10,12

*MRSA indicates methicillin resistant staphylococcus aureus.
